# Systematic review of effectiveness and quality assessment of patient education materials and decision aids for breathlessness

**DOI:** 10.1186/s12890-022-02032-9

**Published:** 2022-06-20

**Authors:** Anthony Paulo Sunjaya, Lexia Bao, Allison Martin, Gian Luca DiTanna, Christine R. Jenkins

**Affiliations:** 1 The George Institute for Global Health, 1 King Street Newtown Missenden Road, P. O. Box M201, Sydney, NSW 2042 Australia; 2 The University of New South Wales, 18 High Street Kensington, Sydney, NSW 2052 Australia; 3 University of British Columbia, Pandosy St, V1Y 1T3, Kelowna, BC 2312 Canada; 4 Concord Clinical School, Concord Repatriation General Hospital, Hospital Road Concord, Sydney, NSW 2139 Australia

**Keywords:** Breathlessness, Shared decision making, Patient education materials, Patient decision aid

## Abstract

**Background:**

Around 10% of adults suffer from clinically significant breathlessness. High quality and actionable patient education materials (PEMs) and patient decision aids (PDAs) have an important role for shared decision making and patient self-management.

**Objective:**

To systematically assess the effectiveness of patient education materials (PEMs) and patient decision aids (PDAs) on clinical outcomes. Secondly, to assess the quality of PEMs and PDAs for breathlessness that are accessible online.

**Methods:**

A systematic review of PEM or PDA intervention for breathlessness published between 1 January 2010 and November 2020 was conducted. An environmental scan and quality assessment of publicly available PEMs and PDAs was also conducted.

**Results:**

Out of 2985 records, five studies were eligible for inclusion in this systematic review. Results of two randomised controlled trials suggest potential effectiveness of PEMs to improve patient reported outcomes and reduce healthcare utilization. In the environmental scan, 88 materials were included. Minimum reading age for most was high (Grade 10) and PEMs scored an average of 87% for understandability and 67% for actionability. Based on the DISCERN tool only 10 were classified as high quality.

**Conclusion:**

There is a paucity of evidence on the effectiveness of PEMs and PDAs for improvement in breathlessness. There is a need to develop higher quality PEMs for breathlessness.

**Supplementary Information:**

The online version contains supplementary material available at 10.1186/s12890-022-02032-9.

## Background

Breathlessness is a common symptom that occurs in many patients with long-term cardiorespiratory conditions such as COPD, asthma, or heart failure [1, 2], as well as many less common conditions, increasing age and physical inactivity, making it often difficult to diagnose causality. Patients with chronic breathlessness often experience reduced functional ability and quality of life, exercise avoidance and heightened anxiety as consequences [3]. Previous studies [4] have found that these patients show limited health seeking behaviour. Poor communication with health practitioners and a lack of helpful written information may contribute to this. On the other hand, previous studies [5] indicated health professionals found breathlessness to be a difficult symptom to discuss with patients due to their inability to provide support, information to patients, and the time it takes to provide a meaningful discussion to this complex symptom.

Health literacy is an important predictor of health outcomes [6]. Currently, patients receive health information from their health professionals during appointments and from published materials both physical and digital, either received through a health professional or found on their own. These patient information sheets and/or webpages together are referred to as patient education materials (PEMs). They have an important role in enhancing the patient’s understanding of their disease and can support shared decision making [6]. PEMs can also support health professionals, being tools that facilitate provision of effective and efficient information on breathlessness to patients, specifically being accessible beyond the clinic setting.

With many different treatment options having varying benefits and limitations, as well as different values of individual patients and families, shared decision making has become important in medical practice. Shared decision making includes the active consideration of patient needs, values and preferences during their own treatment processes. This also helps patients become well informed on treatment options and allows them to actively participate in their own management [7].

Patient decision aids (PDAs) are a form of PEMs designed to help patients participate in decision making for their own health care options. PEMs can be classified as PDAs if they adhere to the qualifying criteria of the International Patient Decision Aid Standards (IPDAS). PDAs can take many forms, varying from online sources such as a webpage or paper-based sources such as factsheets. Studies have shown that PDAs promote shared decision making between patients and health professionals while increasing patient knowledge and reducing passivity during medical consultations [7].

While there are many PDAs available online, high quality and actionable materials are needed for patients to make well-informed decisions. Their readability is crucial to ensure that the materials provided are equitably accessible, especially to those with low health literacy, low technological literacy, low socioeconomic status, and non-English speaking background that are most vulnerable to being breathless and have poor access to care [8]. Even so, two prior Cochrane Systematic Reviews published in 2014 [10] and 2017 [10] on PDAs for any health condition did not include any content relevant for breathlessness. Hence, we conducted a systematic review to answer the research question—In people living with breathlessness associated with a non-malignant disease, do PEMs or PDAs specific for breathlessness [as a sole intervention or as major component of a multimodal intervention] improve health outcomes compared to usual care, standard care or other interventions?

Secondly, we undertook an environmental scan with an aim to perform a quality assessment of the readability, understandability, quality, and actionability of PEMs and PDAs for breathlessness that are accessible online.

## Materials and methods

### Systematic review

A contemporary search was conducted by two of the authors (AS and LB) independently for studies published between 1 January 2010 and November 2020 to ensure that studies included assessed PEMs/PDAs that are still likely to be available and provide evidence-based recommendations that are in line with recent guidelines. This contemporary search also helps reduce heterogeneity when studies are pooled during meta-analysis. The research databases utilised were Cochrane Central Register of Controlled Trials (CENTRAL; latest issue), in the Cochrane Library, Embase Ovid, Pubmed, CINAHL, and PsychInfo. Reference lists of relevant systematic reviews and included studies were also searched to obtain studies of interest. A protocol was developed before the start of the search detailing the full processes of the review including the research question, inclusion/exclusion criteria, search strategy, risk of bias assessment, data extraction, and synthesis, and approved by all authors but was not pre-registered/published. These details, including amendments to the protocol, are presented in Additional file 1: Appendix S1.

#### Population, intervention and comparator

The population of interest were adult patients with breathlessness due to any non-malignant cause. The interventions of interest were PEMs or PDAs for breathlessness and a comparator, either usual care or standard of care or another intervention. PDAs were defined in accordance with the IPDAS as tools designed to help people participate in decision making about health care options when personal preferences are relevant and important [11].

#### Inclusion criteria

- Primary intervention studies of any design published in all languages as we focused on studies that assessed PEM and PDA implementation in practice rather than those that focused only on the development of PEMs or PDAs.
- Studies where PEMs or PDAs provided breathlessness guidance, or that explicitly identified breathlessness as a major part of the PEMs or PDAs’ content, and of the intervention being assessed. Adjuncts to the intervention beyond PEMs or PDAs can be present but should not be the focus of the intervention being assessed.

#### Exclusion criteria

- Studies that assessed PEMs or PDAs that are more disease specific e.g., Asthma Management, COPD management rather than with the goal of managing breathlessness as a symptom.
- Studies that lacked adequate detail on whether management of breathlessness was explicitly included as part of the PEMs or PDAs being assessed.

#### Outcomes

Primary outcomes of interest were improvement in breathlessness as measured by validated scores such as the modified Medical Research Council (mMRC) scale, Borg scale or Dyspnea-12, and improvement in clinically validated scores such as the Hospital Anxiety and Depression Scale (HADS), Chronic Respiratory Disease Questionnaire (CRQ), Asthma Control Questionnaire (ACQ) etc. Other primary outcomes of interest were hospitalisation, mortality, and quality of life. Secondary outcomes of interest included improvements in knowledge, provider and patient satisfaction, health economics analysis, and other externalities. This broad range of impact outcomes was selected as we also aimed to identify which outcomes of interest were utilized by studies and where gaps may remain.

#### Risk of bias assessment

The quality of research studies was assessed in accordance with their study design. Randomized controlled trials were assessed using the Cochrane Risk of Bias v2 tool in accordance with the Cochrane Handbook for Systematic Review of Interventions [12] and observational studies using the Newcastle Ottawa Scale [13].

### Environmental scan and quality assessment

The methods for this study were adapted from previous studies [14] on the assessment of decision aids and are discussed in detail in Additional file 1: Appendix S2. The environmental scan was conducted through a Google search and known online decision aid repositories shown in Additional file 1: Appendix S3.

The study included PEMs and PDAs that address breathlessness as a symptom either independently or as part of a specific disease e.g. COPD, in patients published from 1 January 2010 till 10^th^ November 2020. We classified a material as a patient decision aid if it complied with all qualifying questions in IPDAS v4 [11].

We excluded PEMs and PDAs that are more disease specific e.g. those focusing on Asthma management or COPD management rather than with the goal of managing breathlessness as a symptom. PDAs that required user payment and those developed by companies that sought to market a particular product were also excluded.

Similar to the systematic review process, screening to extraction was done independently by two reviewers (AS and LB) using the open source web tool, Rayyan QRCI [15] and Qualtrics XM. The selection process was recorded and presented in the form of a PRISMA flow diagram.

#### Outcomes

The outcomes of interest were the readability, understandability, actionability and quality of PEMs for breathlessness. Readability was assessed through 7 validated indices—Flesch-Kincaid reading ease index, Flesch-Kincaid grade level, Gunning-Fog score, Coleman-Liau index, SMOG index, Automated readability index and Linsear write formula calculated using an automatic tool (https://readabilityformulas.com). The grade reported by these indices refer to the United States of America educational system. Understandability and actionability were assessed through the Patient Education Materials Evaluation Tool for Print Materials (PEMAT-P) [16]. Quality of materials was assessed using the DISCERN tool [17] and IPDAS v4 [11] criteria.

### Data analysis

We pooled effect sizes (changes from baseline) in the intervention arm by performing Sidik-Jonkman random effects meta-analysis. For studies which did not report the standard deviation of the change we imputed it (using the method suggested by the Cochrane Handbook [18]).

We assessed quantitative heterogeneity by conducting a formal test of homogeneity and evaluating the proportion of variability due to heterogeneity (I^2^). We assessed potential small-study effects by inspecting funnel plot (we report regression-based Egger test *p*-value for completeness).

For studies which were not amenable to pooling, qualitative narrative synthesis was conducted following the Synthesis without meta-analysis (SWiM) guidance [19]. Studies were grouped based on the disease they addressed and priority was provided in reporting results from RCTs compared to observational studies. No transformations were done on the metrics used as we utilised validated measures as our outcomes of interest. Effect estimates were descriptively reported, and study characteristics were reported in tables with their risk of bias results. All descriptive and statistical analyses were performed using Stata 16 (StataCorp LLC, College Station, TX, USA).

## Results

### Systematic review

**Fig. 1:**
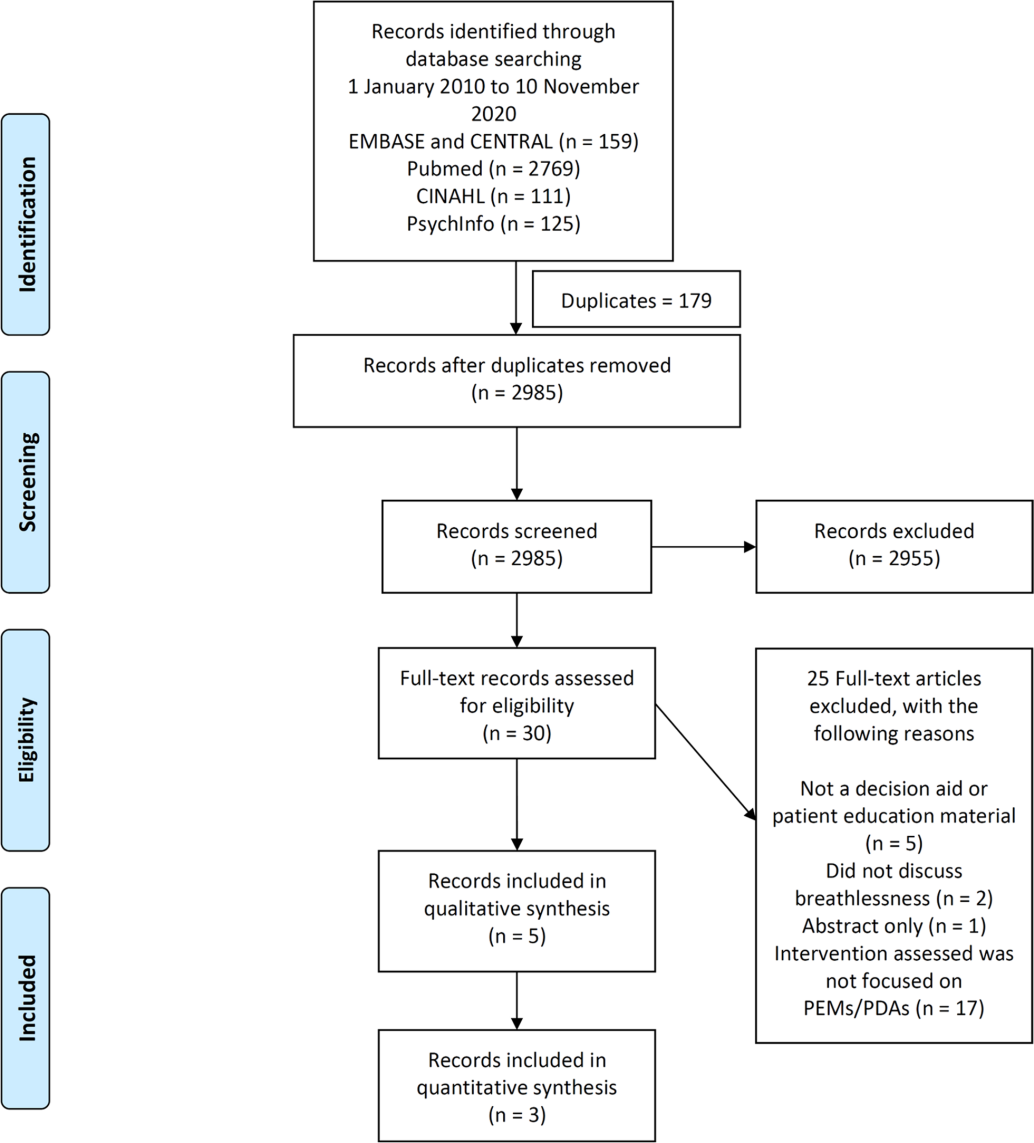

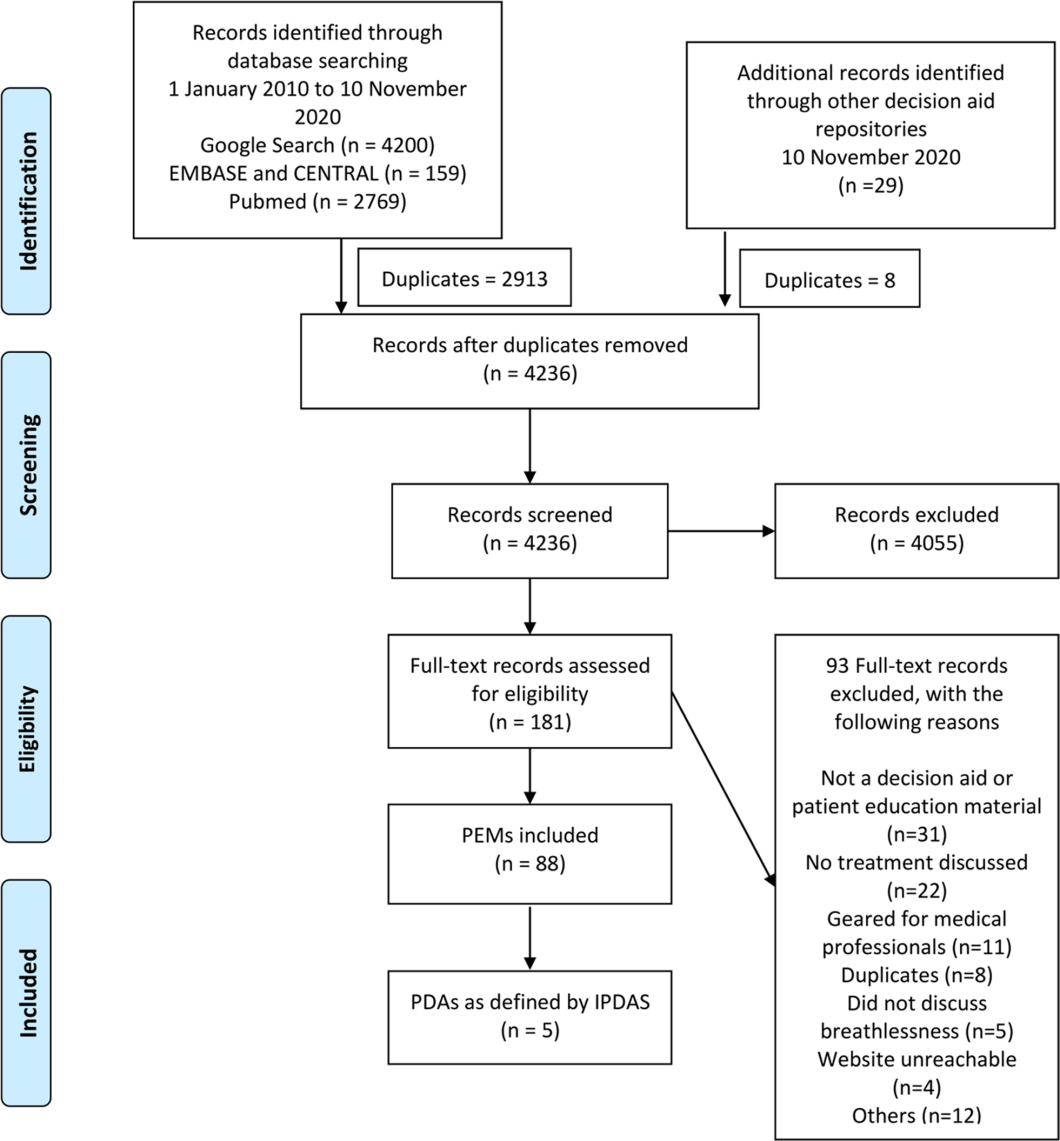
**A** Systematic Review Flow Diagram and **B** PEMs and PDAs Flow Diagram

Of the 2985 records screened, 30 underwent full text review and 5 research studies were included. (Fig. 1A) Studies on remote support such as by Wongpiriyayothar et al. [20], pulmonary rehabilitation such as by Blackstock et al. [21] and breathlessness intervention services such as by Higginson et al. [22] were excluded as while they utilized PEMs or PDAs, they were not the main focus of these studies.

Four of the five were specifically intended for COPD patients. (Table 1) Interventions described in the studies utilised a variety of mediums from brochures to manuals/booklets and videos. In some, PEMs were part of a multifaceted intervention which included provision of hand-held fans and initial face-to-face training. Two RCTs were found—one each for asthma and COPD. Studies were conducted in varied settings and countries including the United Kingdom, Australia, and Saudi Arabia.

**Table 1:** Summary of included research studies

Study	Country	Setting	Design	Participant	Intervention	Comparator	Outcome Measures	RoB
Thomas et al. [23]	UK	Primary Care/General Practice Clinic	RCT (3 arms)	Asthma patients	2 intervention arms: Self-guided intervention (DVD, booklet), Face to face physiotherapist-delivered breathing retraining programme	Usual care	AQLQ (short version)	Low
Howard et al. [24]	UK	Primary Care/General Practice Clinic	RCT	COPD patients	COPD manual including in-person cognitive behavioural intervention	British Lung Foundation Information Booklet	HADS for Anxiety and Depression, CRQ scores	Low
Qian et al. [26]	Australia	Hospital	Pre-Post study	COPD patients with refractory breathlessness despite optimal disease‐directed care, with a mMRC score of 3–4	Breathlessness pack (hand‐held fan, information leaflets and an individualized breathlessness plan) Where applicable, this plan included details regarding the correct use of domiciliary oxygen therapy and/or opioids for breathlessness	None	Dyspnea Severity (mMRC), Quality of life, Qualitative Interviews	High
El-Gendy, [25]	Saudi Arabia	Hospital	Pre-Post study	Male adult COPD patients diagnosed and identified as GOLD stage II and III who were clinically stable	Educational program, using videos and brochures adapted from Saudi guidelines for the diagnosis and management of COPD	None	Dyspnea Severity (not mMRC)	High
Apps et al. [27]	UK	Primary Care/General Practice Clinic	Pre-Post study	COPD patients with a FEV1/FVC ratio of < 70% and mMRC score of 2–5	COPD manual (176 pages) comprising of an exercise program, education topics with goal-setting text, case studies for peer modelling, and activities to encourage problem solving and support behaviour change	None	Dyspnea Severity (not mMRC)	High

*AQLQ asthma quality of life questionnaire, *CTP* coaching by telephone program, *FEV1* forced expiratory volume in 1 s, *FVC* forced vital capacity, *HF-SSS*, *mMRC* modified medical research council breathlessness scale, *NYHA* New York heart association, *RoB* risk of bias

#### Outcomes from randomized controlled trials (RCT)

One three-arm RCT compared the effectiveness of a self-guided intervention, ‘face-to-face’ physiotherapist-delivered breathing retraining program and usual care on quality of life measured using the Asthma Quality of Life Questionnaire (AQLQ) [23]. A statistically significant increase in AQLQ was reported in the self-guided intervention (adjusted mean difference 0.28, 95% confidence interval (CI) 0.11 to 0.44; *p* < 0.001) when compared with usual care. This improvement in AQLQ was reported to be equivalent to the improvement found between the face-to-face physiotherapist delivered breathing retraining program and usual care.

In COPD, a RCT [24] comparing the effectiveness of a COPD manual addressing both physical and mental health with information booklets reported not only an improvement in patient reported outcomes but also found a 42% reduction in total accident and emergency (A&E) and hospital admissions in the COPD manual group compared to a 16% rise in the standard information booklet group.

#### Outcomes from observational studies

A pre-post study [25] in patients with COPD reported a statistically significant (*p* < 0.001) increase in knowledge of COPD and exercises to assist breathing (88%, 80%, and 66% for energy conserving, respiratory muscle exercise, and relaxation techniques respectively) post intervention. The intervention reduced the prevalence of moderate breathlessness from 68 to 48% as measured by the Borg scale. The total number affected also decreased significantly. Marked statistical and clinically meaningful reductions in anxiety also occurred as measured by the HADS.

These results were supported by Qian et al.’s [26] study which included an individualized breathlessness pack (PEM, personalised plan and handheld fan) to participants with severe COPD and refractory breathlessness. After 6 weeks, they reported a clinically significant improvement in breathlessness severity as measured by the Numerical Rating Scale which decreased from 5.6 (SD ± 1.6) to 4.6 (SD ± 2.2).

Another pre-post study in primary care with a COPD self-management manual as an intervention also reported a statistically and clinically significant improvement of 302 s (95% CI 161 to 443; *p* < 0.001) post intervention in the Endurance Shuttle Walking Test (ESWT) results [27].

Three of the five studies, all on COPD patients reported changes to the HADS and CRQ scales. Two were pre-post studies and one RCT, hence only uncontrolled meta-analysis of endpoint-baseline effects (within intervention) was possible as summarised below.

#### HADS scale

**Fig. 2:**
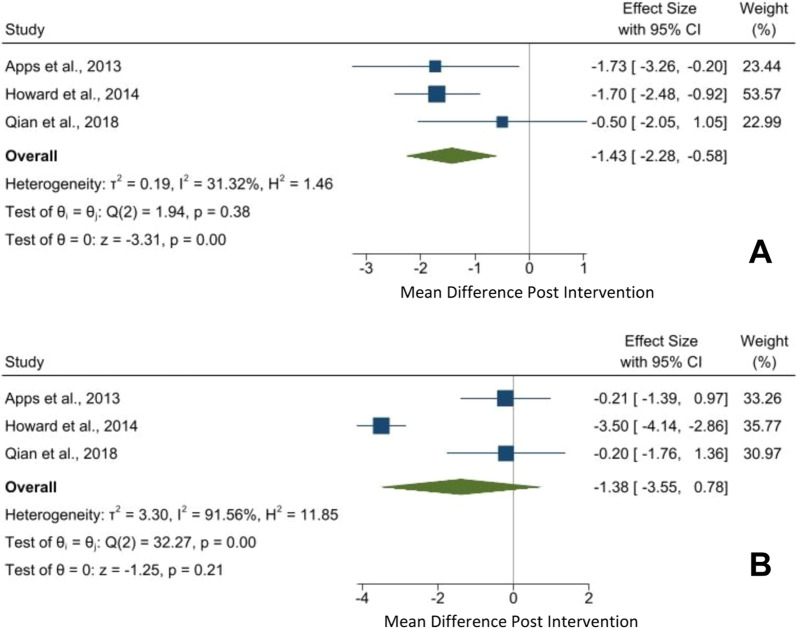
**A** HADS Anxiety and **B** Depression score meta-analysis of within intervention effect

The pooled analysis of 3 studies (n = 175) showed a statistically significant mean reduction in the HADS Anxiety domain by 1.43 (95% CI − 2.28 to − 0.58, *p* =  < 0.01) measured before and after intervention. No statistically significant difference was found for the HADS Depression domain (Mean Difference [MD] 1.38, 95% CI − 3.55 to 0.78, *p* = 0.21). (Fig. 2).

#### CRQ scale

**Fig. 3:**
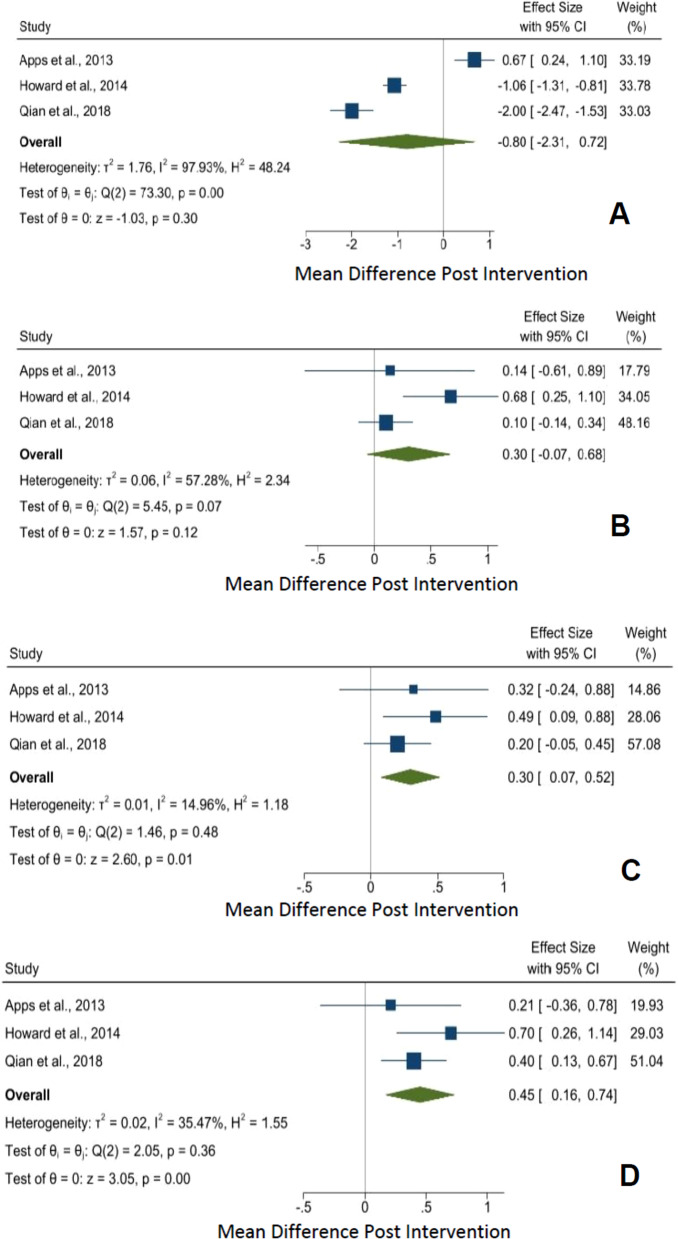
**A** CRQ Dyspnea, **B** CRQ Fatigue, **C** CRQ Emotional and **D** CRQ Mastery score meta-analysis of within intervention effect

Based on 3 studies (n = 175), two of the four CRQ domains—emotional and mastery showed a statistically significant mean increase of 0.30 (95% CI 0.07 to 0.52, *p* = 0.01) and 0.45 (95% CI 0.16 to 0.74, *p* =  < 0.01) respectively measured before and after intervention. However, both the dyspnea and fatigue domains showed no statistically significant mean difference at − 0.80 (95% CI − 2.31 to 0.72, *p* = 0.30) and 0.30 (95% CI − 0.07 to 0.68, *p* = 0.12) respectively. (Fig. 3).

#### Risk of bias

Risk of bias was assessed for all five research studies. Two were found to be of low risk with three at high risk of bias. (Table 1) Eggers test of small study effects showed some evidence of small study effect for HADS Depression. No evidence of small study effects was found for other outcomes. (Additional file 1: Tables S2 and S3).

### Environmental scan and quality assessment

A total of 4236 records were screened after removing duplicates, 181 went into full text review. Eighty-eight PEMs and PDAs were found and analysed – two from research studies and others from publicly available sources. (Fig. 1B).

The majority (51.1%) were for breathlessness followed by hyperventilation (21.6%) and COPD (9.1%). Most were published by hospitals (34.1%) and in the form of printable PDFs (59.1%). Only 5.68% (n = 5) of the records qualified as a patient decision aid (PDA). (Table 2).

**Table 2:** Characteristics of included PEMs and PDAs identified in the Environmental Scan

Publisher Type	N (%)
Hospital	30 (34.1%)
Academic institution	7 (7.9%)
Not for profit organisation	13 (14.8%)
Health professional/medical society	16 (18.2%)
Private company	17 (19.3)
Government	5 (5.7%)
*Continents*	
North America	38 (43.2%)
Europe	35 (39.8%)
Oceania	15 (17%)
*Disease group addressed*	
Breathlessness in general	45 (51.1%)
COPD	8 (9.1%)
Asthma	1 (1.1%)
Heart failure	1 (1.1%)
Cancer	7 (8%)
Psychogenic	4 (4.6%)
Dysfunctional breathing	19 (21.6%)
Others	3 (3.4%)
*Format*	
Static (Webpage format)	35 (39.8%)
Interactive Website	0 (0.00%)
Paper based (printable PDFs)	52 (59.1%)
Video	1 (1.1%)
Other multimedia	0 (0.00%)
*Non-English translation available*	
Yes	1 (1.1%)
No	87 (98.9%)
*Option to reach out for non-English details*	
Yes	14 (15.2%)
No	78 (84.8%)

#### Readability

The composite of the seven readability indices found an average minimum reading level of Grade 10, with 35 PEMs being suitable for the general population (Grade 8), and 1 suitable for low health literacy (Grade 5). The readability consensus showed that the PEMs ranged from “Easy to read” to “Difficult to read”, where most were “Plain English/Standard/Average” (43.18%, n = 38). (Table 3 and Additional file 1: Table S4).

**Table 3:** Summary readability, understandability, actionability and quality assessment of included PEMs and PDAs identified in the environmental scan

	Mean (SD)
*Readability assessment (n = 88)*	
Flesch reading ease (0–100)	60.56 (± 10.38)
Fog scale (0–20)	11.32 (± 2.45)
Flesch-Kincaid grade level (0–18)	11.32 (± 2.45)
Coleman Liau (0–17)	10.51 (± 1.99)
SMOG	8.29 (± 1.70)
Automated readability index (1–14)	9.85 (± 2.83)
Linsear write formula	10.06 (± 3.44)
*Health literacy evaluation (n = 88)*	
PEMAT-understandability	87 (± 10)
PEMAT-actionability	67 (± 23)
*IPDAS (n = 5)*	
Proportion qualified as decision aid	5
Certification criteria	61.5 (± 8.6)
Quality criteria	61.8 (± 13.2)

Quality assessment (DISCERN) (n = 88)	N (%)
Mean (SD)	67 (± 12)
Low	3 (3.41)
Low-moderate	20 (22.73)
Moderate	38 (43.18)
Moderate-high	17 (19.32)
High	10 (11.36)

We also assessed accessibility for those with culturally diverse backgrounds. There was relatively low access for populations with linguistically diverse backgrounds. Out of the 88 PEMs, only 1.14% (n = 1) had non-English translation available (in Spanish), and 15.22% (n = 14) had the option to reach out for non-English details.

**Fig. 4:**
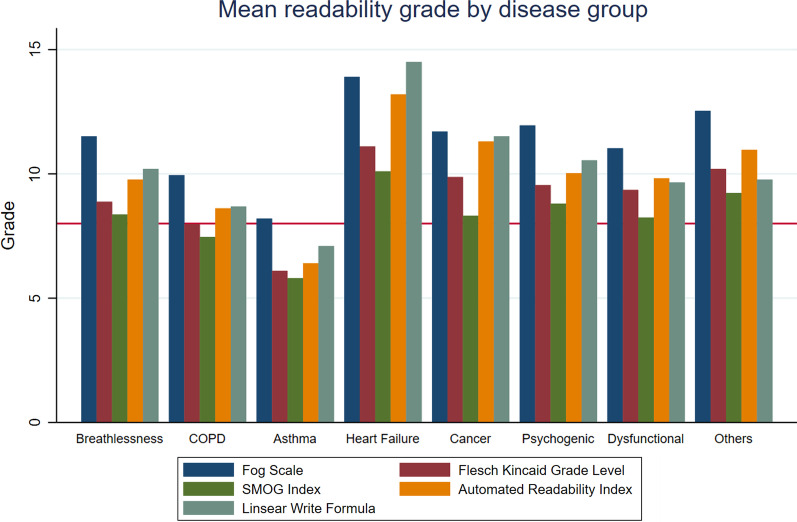
Comparison of readability scores by disease groups. *Grades refers to the number of years of education based on the USA educational system*

When comparing readability scores between disease groups, we found that heart failure PEMs required the highest education level to read, with asthma PEMs being the most readable for the general population (US Grade 8). (Fig. 4).

#### Understandability

Based on our results from PEMAT-P, the mean understandability of the PEMs was 87 (SD ± 7.1) out of a total score of 100. Most PEMs did not include visual aids where they could make the content more easily understood to patients (54.54%, n = 48), and most PEMs did not include any videos (97.72%, n = 86). All PEMs were determined to use common, everyday language, to have informative headers, break the material into short chunks of information. (Table 3 and Additional file 1: Table S4).

#### Actionability

For PEMAT-P, the mean actionability was 61 (SD ± 24.6) out of a total score of 100. Most PEMs (75%, n = 66) did not include a tangible tool such as menu planners and checklists, but most PEMs explained how to use visual aids to take action (82.95%, n = 53). Most materials clearly identified at least one action the reader can take (97.73%, n = 86), and most addressed the reader directly when describing actions (96.59%, n = 85). (Table 3 and Additional file 1: Table S4).

**Fig. 5:**
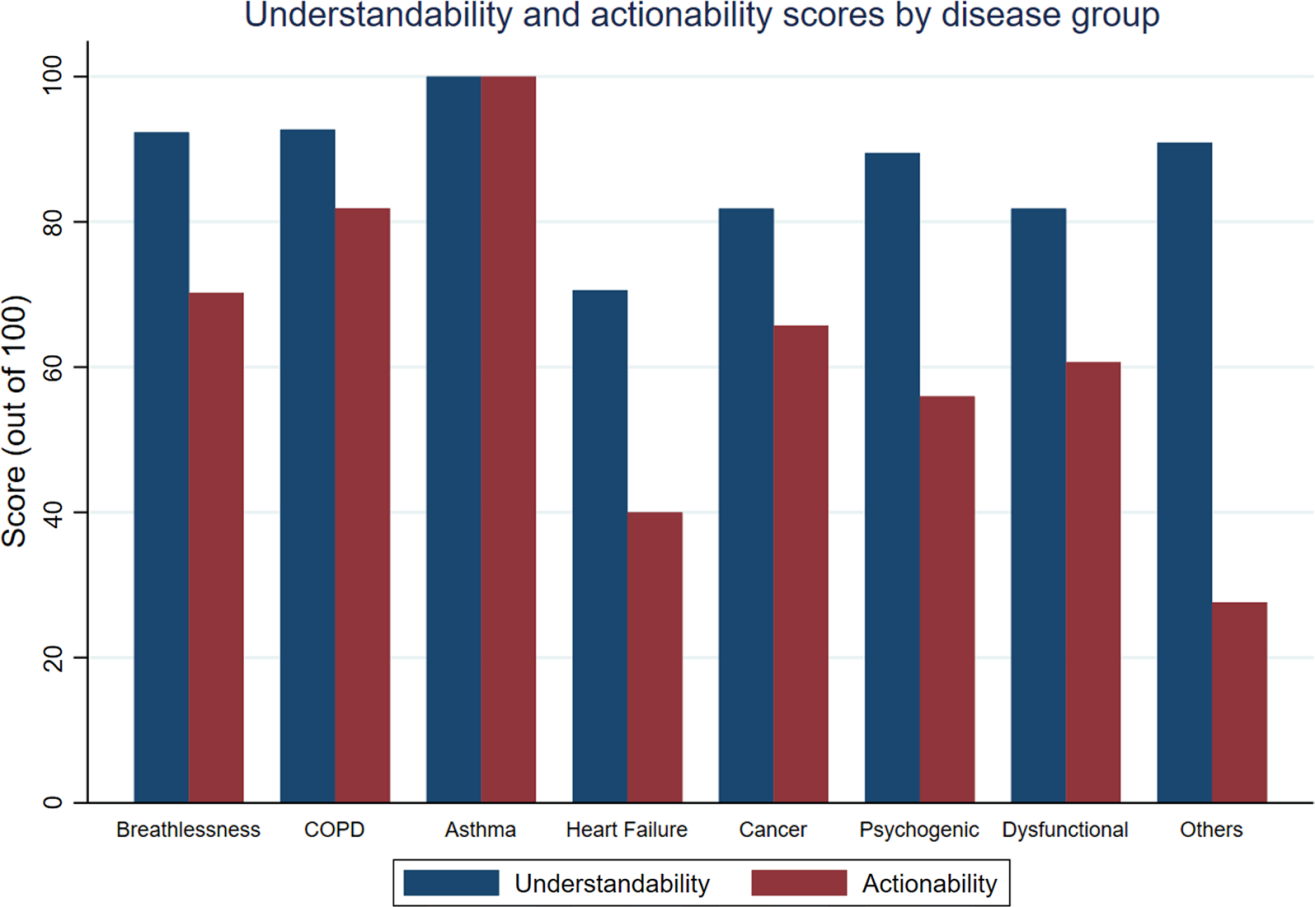
Comparison of understandability and actionability scores by disease groups

As shown in Fig. 5, a comparison of PEMs based on their disease groups found asthma PEMs to be most understandable and actionable. Heart failure and cancer PEMs were found to be on average the least understandable and actionable, respectively.

#### Quality assessment using DISCERN

Based on DISCERN, the overall quality of 43.18% (n = 38) of the PEMs was determined to be “Moderate (Potentially important but not serious shortcomings)”. 3.41% (n = 3) were determined to be of low quality, while 11.36% (n = 10) were high quality. Most PEMs did not report the sources of information that were used (62.50%, n = 55), when the information used was produced (71.59%, n = 63), describe the risks of each treatment (73.86%, n = 65), describe what would happen if no treatment is used (73.86%, n = 65), or describe how the treatment choices affect the overall quality of life (51.13%, n = 45). However, most PEMs are balanced and unbiased (79.54%, n = 70), provide details of additional sources of support and information (53.41%, n = 47), offer details on more than one possible treatment choice (77.27%, n = 68), and provide support for shared decision-making (45.45%, n = 40). (Additional file 1: Table S4).

Of the 88 PEMs, only 5.68% (n = 5), fulfilled all the IPDAS certification criteria [11] as a decision aid. 68.18% (n = 60) of the PEMs did not describe the positive benefits of each option that were provided, and 65.91% (n = 58) did not explicitly state the decision that needs to be considered. When the five PDAs were assessed against the IPDAS certification and quality criteria, we found none reported a needs assessment with health professionals during the PDA development process. As well, none allowed the user to compare outcome probabilities across options using the same time period or using the same denominator. Four however did include tools like worksheets/lists of questions to use when discussing options with a practitioner. (Additional file 1: Table S5).

## Discussion

In the systematic review of published intervention study, the five identified studies all concluded that the use of PEMs in patient care led to significant improvement in breathlessness outcomes compared to patients who did not receive these materials. Some have observed improvements in patients’ reported outcomes, such as breathlessness, depression, and anxiety. Statistically significant differences were found in several pooled patient-reported outcomes namely – HADS Anxiety, CRQ Emotional and CRQ Mastery. One RCT by Howard et al. [24] in COPD patients reported significant reduction in patients’ utilization of various health-care services post intervention. We note that in this study, an educational session was conducted when the PEM was provided, with two further remote follow ups by phone.

Even so, the one RCT [23] and two pre-post studies [25, 27] that only provided PEMs as an intervention reported significant improvements in breathlessness severity and exercise endurance post-intervention. The three-arm RCT [23] in asthma patients was also able to show the equivalence of the PEM intervention against a face-to-face physiotherapist-delivered breathing retraining program.

In contrast, the control arm of one RCT [24] which provided informational brochures did not result in any improvement in patient reported outcomes and instead increased total visits to the ED and days in hospital, although the intervention arm which provided a manual that addressed both the physical and mental health aspect of the disease with a one day CBT workshop for its implementation, resulted in significantly reduced healthcare use. This study suggests that not all PEMs are equally useful and that training, even just an hour at initiation, is of importance to ensure tools are used properly and result in positive outcomes. The addition of other adjuncts such as routine face-to-face visits and remote coaching by telephone may enhance this benefit even further as shown in a recent Cochrane Systematic Review [28, 29]. However, from a scalability perspective, our review showed that in multiple settings, the provision of high-quality PEMs is able to provide clinical benefits, especially to COPD patients.

Our environmental scan identified 88 PEMs and PDAs that were publicly accessible. Only 5 PEMs fulfilled the IPDAS criteria to qualify as a PDA. The 5 PEMs that fulfilled the initial IPDAS qualifying criteria, only met a median of 30% of the quality criteria. Therefore, even the PEMs on breathlessness that qualified as PDAs were found to be of low quality as decision aids. There is a need to develop reliable and high-quality decision aids for patients and carers. This observation is not unique to breathlessness and was also similarly reported in a previous review of decision aids for atrial fibrillation, another common disease in practice [30].

From the 88 PEMs that were assessed for quality and content, the readability indices using a broad range of validated tools showed that the average minimum reading level was Grade 10, and only one PEM was suitable for those with low health literacy (Grade 5). Breathlessness is often present in the elderly and increases in prevalence with ageing. However, studies [31, 32] have shown that elderly patients (who usually have the most comorbidities) are more likely to lack the literacy skills and digital competency needed to function adequately in the current healthcare environment.

PEMs written at a level too high for the average adult may increase the risk of promoting health disparities as shown by Sentell et al. [33]. However, a recent review by Yen et al. showed that the use of patient decision aids in socially disadvantaged populations has the potential to improve patient outcomes [34]. Hence it is of concern that our results show that breathlessness PEMs are written at a level too high for the average population. PEMs written at too high a literacy level are not only unlikely to support better outcomes but may contribute to increased health disparity, particularly affecting those with low literacy and low health literacy.

In addition, we also found only one PEM had non-English translations available, and only 15.22% (n = 14) offered the option to reach out for non-English details. Therefore, patients from culturally and linguistically diverse backgrounds may be at a disadvantage in understanding their treatment options, risk profile and receiving care that is in accordance to their values.

Based on the quality assessment conducted using the DISCERN tool, only 45.45% (n = 40) PEMs provided support for shared decision making, and 73.86% (n = 63) did not report the risks associated with each management option. This highlights a significant deficiency for currently available PEMs for breathlessness. As PEMs may be used to promote shared decision making between patients and their health professionals or family, it is important that they list the benefits and consequences of each treatment option explicitly and comprehensively, to enable the patient to understand each treatment option and make well-informed decisions.

## Limitations

The five research studies that were analysed were mostly at the pilot phase and have a high risk of bias, hence these results must be interpreted with caution. There was some evidence of small study effects for only HADS Depression. However, we acknowledge that there were only three studies as part of this analysis, all undertaken in COPD patients. Studies also mostly had small sample sizes with high likelihood of selection bias.

Due to the sparse evidence, we were only able to pool the effect sizes of the intervention arms and not of controlled results. Hence, we were unable to ascertain whether the placebo or Hawthorne effect may have played a role in these results. High heterogeneity was found between studies for several outcomes which may be due to the difference in the country and setting where the studies took place, and difference in the content of interventions assessed. Even for outcomes with low heterogeneity, the small number of studies available to pool and their small sample sizes may potentially underestimate the difference in effects between studies. We also note that for two of the included studies PEMS were part of a multifaceted intervention, and it was not possible to ascertain the contribution of PEMs to the overall impact. Determining the true effectiveness of PEMs on patient healthcare outcomes will require further studies.

For the environmental scan, our search methodology was conducted using English keywords, which could influence the results of our search. For future studies, it would be important to conduct similar searches using keywords from other languages as well.

In conclusion, our findings suggest potential benefits of using PEMs and PDAs to improve care in patients with breathlessness, although our analysis also included studies where PEMs and PDAs were used as part of a multifaceted intervention rather than the sole intervention. We found deficiencies in the information describing the benefits and risks associated with each treatment option, making shared decision making more difficult. The results also highlight the current scarcity of PEMs and PDAs that are written at the recommended reading age of Grade 8, which would help to ensure the information provided is accessible by people of all socioeconomic, cultural, and linguistic groups. Health and social inequalities will be magnified if major population groups are unable to access materials and make well-informed decisions on their own health and well-being. Those people who are the least healthy, least knowledgeable, and least engaged would be likely be able to gain the most benefit when materials are developed in a way that is inclusive and easily accessed. Our study suggests there is a still a great deal to achieve in this regard.

## Supplementary Information

**Additional file 1:** Manuscript Supplement.

## Data Availability

The dataset supporting the conclusions of this article is included within the article and its Additional file 1.

## Abbreviations

A&E: Accident and emergency
ACQ: Asthma control questionnaire
AQLQ: Asthma quality of life questionnaire
CENTRAL: Cochrane central register of controlled trials
CRQ: Chronic respiratory disease questionnaire
COPD: Chronic obstructive pulmonary disease
HADS: Hospital anxiety and depression scale
IPDAS: International patient decision aid standards
mMRC: Modified medical research council
PDA: Patient decision aids
PEM: Patient education materials
PEMAT-P: PEM evaluation tool for print materials
RCT: Randomized controlled studies
