# Supplementary material for: Systematic review of effectiveness and quality assessment of patient education materials and decision aids for breathlessness
Source: BMC Pulm Med. 2022 Jun 20;22:237. doi: 10.1186/s12890-022-02032-9 (PMC9208236; doi:10.1186/s12890-022-02032-9)
Supplement: Supplementary file 1 — Additional file 1: Manuscript Supplement. [file 12890_2022_2032_MOESM1_ESM.docx]

**ADDITIONAL FILE 1**

**Systematic Review of Effectiveness and Quality Assessment of Patient Education Materials and Decision Aids for Breathlessness**

*Anthony Paulo Sunjaya, Lexia Bao, Allison Martin, Gian Luca Di Tanna, Christine Jenkins*

Appendix S1. Systematic Review Search strategy and Methods

### Search strategy and selection criteria

A contemporary search was conducted by two of the authors (AS and LB) independently for studies published between 1 January 2010 to November 2020. The research databases utilised were Cochrane Central Register of Controlled Trials (CENTRAL; latest issue), in the Cochrane Library, Embase Ovid, Pubmed, CINAHL, and PsychInfo using keywords as presented below.

**Database serving as example:** Embase Ovid

| **Concept** | **Keywords, Synonyms** | **Subject Headings [MeSH]** | **Terms included under the MeSH** |
| --- | --- | --- | --- |
| **Breathlessness** | short* of breath.mp.  air hunger.mp.  Breathlessness*  difficult* breathing  breathing difficult*  dyspne*  hyperventilation | exp dyspnea/  exp hyperventilation/ | breathing difficulties  breathing difficulty  breathlessness  difficult breathing  difficult respiration  difficulty breathing  dyspneas  dyspneic syndrome  dyspnoea  dyspnoeae  dyspnoeas  labored respiration  laboured respiration  lung dyspnea  lung dyspnoea  shortness of breath |
| **Patient Education Materials and Patient Decision Aids** | patient decision aid.mp.  Patient decision tool  Patient decision aid  Patient fact sheet  Patient information  Patient brochure  patient information.mp. | exp patient information/ | information |

| exp dyspnea/  short* of breath.mp.  exp hyperventilation/  air hunger.mp.  (Breathlessness* or difficult* breathing or breathing difficult* or dyspne* or hyperventilation).mp. [mp=ti, ot, ab, sh, hw, kw, tn, dm, mf, dv, fx, dq]  1 or 2 or 3 or 4 or 5  patient decision aid.mp.  (Patient decision tool or Patient decision aid or Patient fact sheet or Patient information or Patient brochure).mp. [mp=ti, ot, ab, sh, hw, kw, tn, dm, mf, dv, fx, dq]  patient information.mp. or exp patient information/  7 or 8 or 9  6 and 10  limit 11 to yr="2010 -Current" |
| --- |

### Population, Intervention and Comparator

The population of interest were adult patients with breathlessness due to any non-malignant cause. The interventions of interest were PEMs or PDAs for breathlessness and a comparator, either usual care or standard of care or another intervention. PDAs were defined in accordance with the International Patient Decision Aid Standards (IPDAS) as tools designed to help people participate in decision making about health care options when personal preferences are relevant and important.[1]

Inclusion criteria:

- Primary intervention studies of any design published in all languages as we focused on studies that assessed PEM and PDA implementation in practice rather than those that focused only on the development of PEMs or PDAs.
- Studies where PEMs or PDAs provided breathlessness guidance, or that explicitly identified breathlessness as a major part of the PEMs or PDAs’ content, and of the intervention being assessed. Adjuncts to the intervention beyond PEMs or PDAs can be present but should not be the focus of the intervention being assessed.

Exclusion criteria:

- Studies that assessed PEMs or PDAs that are more disease specific e.g., Asthma Management, COPD management rather than with the goal of managing breathlessness as a symptom.
- Studies that lacked adequate detail on whether management of breathlessness was explicitly included as part of the PEMs or PDAs being assessed.

### Outcomes

Primary outcomes of interest were improvement in breathlessness as measured by validated scores such as the modified Medical Research Council (mMRC) scale, Borg scale or Dyspnea-12, and improvement in clinically validated scores such as the Hospital Anxiety and Depression Scale (HADS), Chronic Respiratory Disease Questionnaire (CRQ), Asthma Control Questionnaire (ACQ) etc. Other primary outcomes of interest were hospitalisation, mortality, and quality of life. Secondary outcomes of interest included improvements in knowledge, provider and patient satisfaction, health economics analysis, and other externalities.

### Data collection

All titles and abstracts retrieved by electronic searching of the databases were downloaded to a reference manager where duplicates were identified and removed (Endnote X9 for Windows, Thomson Reuters, Philadelphia, USA). Screening was done independently by two reviewers (AS and LB) using the open source web tool, Rayyan QRCI[2]. Full text articles that were screened and included were then downloaded. Two reviewers (AS and LB) independently screened the full text against the inclusion and exclusion criteria, reasons were documented for excluding studies. All disagreements were resolved by discussion, or if required the outcome was decided by a third reviewer (AM or CJ). The selection process was recorded and presented in the form of a PRISMA flow diagram.

Data extraction was conducted using a standard electronic form (Qualtrics XM). Data extracted included characteristics of study participants, interventions, comparators, outcomes and those required for quality assessment. The quality of research studies was assessed in accordance with their study design. Randomized controlled trials were assessed using the Cochrane Risk of Bias v2 tool in accordance with the Cochrane Handbook for Systematic Review of Interventions[3] and observational studies using the Newcastle Ottawa Scale[4]. Quality assessment and data extraction were done by any one of the reviewers (AS and LB) and then independently checked by another reviewer (AS and LB). Any disagreements were resolved by consensus or by a third reviewer (AM or CJ).

## Data Analysis

We pooled effect sizes (changes from baseline) in the intervention arm by performing Sidik-Jonkman random effects meta-analysis. For studies which did not report the standard deviation of the change we imputed it (using the method suggested by the Cochrane Handbook [5]).

We assessed the quantitative heterogeneity by conducting a formal test of homogeneity and evaluating the proportion of variability due to heterogeneity (I^2^). We assessed potential small-study effects by inspecting funnel plot (we report regression-based Egger test p-value for completeness).

For studies which were not amenable to pooling, qualitative narrative synthesis was conducted following the Synthesis without meta-analysis (SWiM) guidance.[19] Studies were grouped based on the disease they addressed and priority was provided in reporting results from RCTs compared to observational studies. No transformations were done on the metrics used as we utilised validated measures as our outcomes of interest. Effect estimates were descriptively reported, and study characteristics were reported in tables with their risk of bias results. All descriptive and statistical analyses were performed using Stata 16 (StataCorp LLC, College Station, TX, USA).

## Amendments to the review protocol

- The word “comprehensive search” has been revised to “contemporary search” to reflect the focus of our search on recent studies and acknowledged that we did not conduct an exhaustive search of all databases.
- Additional databases (CINAHL and PsychInfo) were searched following recommendations from journal reviewers and discussion among all co-authors.
- Revisions were made to the wording to clarify the eligibility criteria of the systematic review following recommendations from journal reviewers and discussion among all co-authors.
- The data analysis section was expanded to include references to imputation of standard deviation (as suggested by the Cochrane Handbook) as not all studies provided this detail and further details were provided with regards to the qualitative synthesis conducted.

Appendix S2. Environmental Scan and Quality Assessment Search Strategy and Methods

The methods for this study were adapted from previous researches[6] on the assessment of decision aids.

### Search strategy and selection criteria

The environmental scan was conducted through a Google search and known online decision aid repositories shown in Appendix S3. For the Google search, the first 50 search results for each unique combination of search terms not including web advertisements were exported using the Scraper plugin in Google Chrome[7]) into a spreadsheet. Duplicates of the search results were removed. Similarly, relevant patient education materials and patient decision aids in known repositories listed were added into the spreadsheet and duplicates removed. When PEMs and PDAs from the research studies were available, we also included them for quality assessment.

The study included patient education materials and patient decision aids that address breathlessness as a symptom either independently or as part of a specific disease eg in COPD patients published from 1 January 2010 till 10^th^ November 2020. Patient decision aids are defined in accordance to the International Patient Decision Aid Standards (IPDAS) as tools designed to help people participate in decision making about health care options when personal preferences are relevant and important.[1] We classified a material as a patient decision aid if it complied with all qualifying questions in IPDAS v4[1].

We excluded PEMs and PDAs that are more disease specific eg those focusing on Asthma management or COPD management rather than with the goal of managing breathlessness as a symptom. PDAs that are paid material and those developed by companies that sought to market a particular product were also excluded.

### Data collection

Similar to the systematic review process, screening was done independently by two reviewers (AS and LB) using the open source web tool, Rayyan QRCI[2]. Webpages and digital materials of all studies that were screened and included were then downloaded. Two reviewers (AS and LB) independently screened the materials against the inclusion and exclusion criteria, reasons were documented for excluding studies. The selection process was recorded and presented in the form of a PRISMA flow diagram.

Data extraction was conducted using a standardised electronic form using Qualtrics XM. Data extracted include details on the author of the decision aids, its format (interactive, static, paper-based, diseases addressed and access for linguistically diverse background. Furthermore, the required details for outcome assessments were collected.

Quality assessment and data extraction were done by any one of the reviewers (AS and LB) and then independently checked by another reviewer (AS and LB). Any disagreements were resolved by consensus or by a third reviewer (AM or CJ).

### Outcomes

The outcomes of interest were the readability, understandability, actionability and quality of patient education materials for breathlessness.

*Readability*

Readability was assessed by 7 validated indices calculated using an automatic tool (https://readabilityformulas.com) which has been utilised in a previous study to assess readability of online materials.[8] The 7 validated indices utilised to generate the composite score were - Flesch-Kincaid reading ease index, Flesch-Kincaid grade level, Gunning-Fog score, Coleman-Liau index, SMOG index, Automated readability index and Linsear write formula.

*Understandability and Actionability*

Understandability and actionability were assessed through the Patient Education Materials Evaluation Tool for Print Materials (PEMAT-P). The understandability domain assesses how consumers of diverse background and health literacy can process and explain key messages in a material. Whereas actionability refers to how consumers of diverse background and health literacy can identify points of action from the material presented. Each domain consists of several questions with yes, no, or not applicable as possible answers. Scores were calculated as a percentage of the number of questions marked as yes compared with the total number of questions assessed except those noted as not applicable.[9]

*Quality*

The quality of all materials was assessed using the DISCERN tool[10], a tool designed to assess the quality of written information about treatment choices based on 15 questions across 2 domains - is the publication reliable; and how good is the quality of information on treatment choices, with an overall rating of the publication. (Table S1)

Table 1. DISCERN Quality Assessment Classification

| High | Yes or partially yes to all questions |
| --- | --- |
| Moderate-High | 2-5 were partial or less than 2 questions were no or partially no |
| Moderate | between 2 to 6 questions were no/partially no or if the majority were partial |
| Low-Moderate | 7 to 10 questions were no or partially no |
| Low | greater than 10 questions were no or partially no |

For patient decision aids, additional quality assessments were conducted to assess concordance with the IPDAS v4[1] criteria certification and quality domains. The certification criteria relate to quality of the evidence synthesis process, open disclosure of funding sources, and a balanced presentation of options whereas the quality criteria relate to items that were desirable but not essential for reducing harmful bias.

## Data Analysis

All descriptive analyses were performed using Stata 16 (StataCorp LLC, College Station, TX, USA).

Appendix S3. List of Patient Education Material Repositories

| **Organisation** | **Website** |
| --- | --- |
| **The Decision Aid Library Inventory (DALI) – Ottawa Research Institute** | [**https://decisionaid.ohri.ca/AZlist.html**](https://decisionaid.ohri.ca/AZlist.html) |
| **Option grids** | [**http://optiongrid.org/**](http://optiongrid.org/) |
| **Agency for Healthcare Research and Quality** | https://www.innovations.ahrq.gov/browse-by-subject |
| **NHS (accessible)** | <https://www.england.nhs.uk/shared-decision-making/guidance-and-resources/> |
| **NICE Decision Aids** | https://www.evidence.nhs.uk/search?om=%5b%7b%22ety%22:%5b%22Patient%20Decision%20Aids%22%5d%7d,%7b%22srn%22:%5b%22National%20Institute%20for%20Health%20and%20Care%20Excellence%20-%20NICE%22%5d%7d%5d |
| **Mayo Clinic Decision Aids** | [**http://www.mayoclinic.org/**](http://www.mayoclinic.org/) |
| **MAGIC SHARE-IT Public Guidelines/Decision Aids** | [**https://www.magicapp.org/app#/guidelines**](https://www.magicapp.org/app) |
| **Decision Boxes at Laval University** | https://www.boitedecision.ulaval.ca/en/ |
| **CeMPED Decision Aids at Sydney University** | [**http://www.psych.usyd.edu.au/cemped/com_decision_aids.shtml**](http://www.psych.usyd.edu.au/cemped/com_decision_aids.shtml) |
| **Health Fact Boxes at the Harding Centre for Risk Literacy** | https://www.hardingcenter.de/en/fact-boxes |
| **Patient Decision Aids site (mostly NHS, OG, M)** | [**http://patient.info/decision-aids**](http://patient.info/decision-aids) |
| **Severe Asthma Center for Research Excellence** | https://toolkit.severeasthma.org.au/co-morbidities/pulmonary-upper-airways/dysfunctional-breathing/ |
| **Chest Foundation** | https://foundation.chestnet.org/ |
| **Asthma Australia** | https://asthma.org.au/what-we-do/how-we-can-help/resources/#B |
| **Lung Foundation Australia** | https://lungfoundation.com.au/ |
| **Heart Foundation Australia** | https://www.heartfoundation.org.au/ |
| **European Lung Foundation** | https://www.europeanlung.org/en/lung-disease-and-information/factsheets/english/ |
| **British Lung Foundation** | https://www.blf.org.uk/ |
| **American Lung Association** | https://www.lung.org/ |
| **HealthDirect** | https://www.healthdirect.gov.au/ |
| **WebMD** | https://webmd.com |
| **MedlinePlus** | https://medlineplus.gov/ |
| **Merck Patient Manuals** | https://www.msdmanuals.com/ |
| **UHN Patient Education** | http://www.uhnpatienteducation.ca/ |
| **British Thoracic Society / Primary Care Respiratory Society UK** | https://www.respiratoryfutures.org.uk/resources |

**Table 2.** Study Quality Assessment

Risk of Bias Assessment using the Newcastle Ottawa Scale

| **Study** | **Selection** | **Comparability** | **Exposure** | **Total Stars** | **Risk of Bias** |
| --- | --- | --- | --- | --- | --- |
| El-Gendy[11] | ☆ | - | ☆☆ | 3 | High |
| Qian et al[12] | ☆ | - | ☆☆ | 3 | High |
| Apps et al[13] | ☆ | - | ☆☆ | 3 | High |

Risk of Bias Assessment using the Cochrane Risk of Bias tool v2

| **Study** | **Randomization** | **Deviation from Intended Interventions** | **Missing Outcome Data** | **Measurement** | **Reporting** | **Risk of Bias** |
| --- | --- | --- | --- | --- | --- | --- |
| Howard et al., 2014[15] | Low | Low | Low | Low | Low | Low |
| Thomas et al., 2017[16] | Low | Low | Low | Low | Low | Low |

**Table 3.** *Assessment of Publication Bias*

| **Outcome** | **Egger’s Test (P-value)** |
| --- | --- |
| HADS Anxiety | 0.5194 |
| HADS Depression | 0.0272 |
| CRQ Dyspnea | 0.9996 |
| CRQ Fatigue | 0.9218 |
| CRQ Emotional | 0.5264 |
| CRQ Mastery | 0.8844 |

**Table 4.** Summary of Included Patient Education Materials (n=88)

| ID | PEMAT-P | | DISCERN^&^ | Readability indices^#^ | | | | | | |
| --- | --- | --- | --- | --- | --- | --- | --- | --- | --- | --- |
|  | (0-100) | |  | Mean (SD) | | | | | | |
|  | Understand | Action |  | FRE | Fog scale | Fle | Col | SMOG | ARI | Lin |
|  |  |  |  | (0–100) | (0-20) | (0-18) | (0-17) |  | (1-14) |  |
| PEM_01[17] | 100.00 | 83.33 | 4 | 63.3 | 12.6 | 9.6 | 10 | 8.7 | 10.9 | 12.7 |
| PEM_02[18] | 81.82 | 40.00 | 2 | 65.5 | 11 | 7.3 | 10 | 8.3 | 7.5 | 7.4 |
| PEM_03[19] | 100.00 | 83.33 | 3 | 61.8 | 10.1 | 8.5 | 10 | 8.6 | 8.9 | 8.9 |
| PEM_04[20] | 83.33 | 50.00 | 3 | 53 | 12.5 | 10.1 | 11 | 9.2 | 10.3 | 10.9 |
| PEM_05[21] | 86.67 | 80.00 | 3 | 55.1 | 13.6 | 11 | 11 | 10.2 | 12.3 | 13.9 |
| PEM_06[22] | 70.59 | 40.00 | 3 | 56.3 | 13.9 | 11.1 | 11 | 10.1 | 13.2 | 14.5 |
| PEM_07[23] | 86.67 | 83.33 | 2 | 70.3 | 7.9 | 5.6 | 10 | 6 | 5.1 | 4.3 |
| PEM_08[24] | 94.12 | 83.33 | 3 | 68.7 | 11 | 8.6 | 10 | 8.3 | 10.4 | 11.9 |
| PEM_09[25] | 86.67 | 83.33 | 3 | 61.7 | 11.5 | 8.9 | 11 | 8.3 | 10.1 | 10.6 |
| PEM_10[26] | 94.44 | 85.71 | 5 | 66.8 | 9.2 | 7 | 9 | 7 | 6.6 | 6.6 |
| PEM_11[27] | 80.00 | 50.00 | 3 | 55 | 14.3 | 11.5 | 10 | 9.8 | 13 | 14.8 |
| PEM_12[28] | 94.12 | 100.00 | 4 | 68.9 | 8.4 | 7.8 | 10 | 6 | 8.9 | 8.3 |
| PEM_13[29] | 75.00 | 60.00 | 3 | 43.8 | 15.9 | 13.3 | 12 | 11.2 | 14.6 | 16.1 |
| PEM_14[30] | 84.21 | 83.33 | 3 | 73.5 | 8 | 6.7 | 10 | 5.3 | 8.5 | 7.2 |
| PEM_15[31] | 66.67 | 40.00 | 2 | 60.8 | 9.8 | 9.5 | 10 | 7.6 | 10.3 | 11.3 |
| PEM_16[32] | 87.50 | 66.67 | 3 | 77.3 | 7.9 | 5.5 | 9 | 5.8 | 6.1 | 6.1 |
| PEM_17[33] | 92.31 | 60.00 | 3 | 60.2 | 12.3 | 7.8 | 13 | 8.8 | 9.1 | 7.2 |
| PEM_18[34] | 94.12 | 66.67 | 3 | 51.8 | 12.1 | 10.3 | 13 | 8.9 | 12.1 | 11 |
| PEM_19[35] | 81.82 | 40.00 | 3 | 60.1 | 12 | 9.5 | 10 | 9.3 | 10.1 | 11.9 |
| PEM_20[36] | 90.91 | 60.00 | 2 | 66.6 | 11 | 8.9 | 10 | 7.2 | 10.9 | 11.4 |
| PEM_21[37] | 88.24 | 85.71 | 4 | 68 | 10.9 | 7.9 | 9 | 7.8 | 8.4 | 9 |
| PEM_22[38] | 94.12 | 83.33 | 3 | 72.9 | 9.3 | 6.2 | 9 | 7.1 | 6 | 6.8 |
| PEM_23[39] | 76.92 | 60.00 | 3 | 50.1 | 13 | 11.2 | 12 | 10.1 | 12.1 | 12.9 |
| PEM_24[40] | 100.00 | 42.86 | 5 | 60.8 | 11.4 | 8.9 | 10 | 8.6 | 9.2 | 9.5 |
| PEM_25[41] | 66.67 | 60.00 | 1 | 31.8 | 20.4 | 18.3 | 13 | 13.5 | 22.2 | 24.7 |
| PEM_26[42] | 75.00 | 60.00 | 3 | 34.4 | 14.8 | 12.4 | 14 | 11.2 | 12.1 | 11.6 |
| PEM_27[43] | 63.64 | 60.00 | 4 | 50.2 | 14.7 | 10.1 | 13 | 10.6 | 10.9 | 11.1 |
| PEM_28[44] | 81.82 | 80.00 | 2 | 35.4 | 16.4 | 13.1 | 17 | 11.9 | 16.2 | 13.9 |
| PEM_29[45] | 81.82 | 60.00 | 2 | 69.3 | 8.2 | 7.1 | 9 | 6 | 7.2 | 7.1 |
| PEM_30[46] | 84.62 | 66.67 | 3 | 71.5 | 9.9 | 7.4 | 9 | 6.8 | 8.2 | 8.7 |
| PEM_31[47] | 93.75 | 100.00 | 5 | 52.3 | 12.5 | 10.4 | 12 | 10.3 | 11.2 | 12.1 |
| PEM_32[25] | 100.00 | 50.00 | 3 | 72.4 | 7.5 | 5.6 | 11 | 6 | 6.4 | 5 |
| PEM_33[48] | 72.73 | 60.00 | 2 | 71.3 | 9 | 5.9 | 10 | 6.7 | 5.8 | 5.4 |
| PEM_34[49] | 91.67 | 75.00 | 3 | 65.1 | 12.4 | 10 | 8 | 8.1 | 10.8 | 13.7 |
| PEM_35[50] | 75.00 | 40.00 | 3 | 41.5 | 13.4 | 11.7 | 14 | 10 | 12.4 | 11.5 |
| PEM_36[51] | 88.24 | 100.00 | 3 | 59 | 11.7 | 8.8 | 12 | 8.7 | 9.7 | 8.8 |
| PEM_37[52] | 92.86 | 40.00 | 2 | 48.4 | 14.2 | 12.4 | 12 | 10.7 | 14.5 | 15.2 |
| PEM_38[53] | 100.00 | 100.00 | 1 | 70.8 | 9.8 | 7.9 | 10 | 6.9 | 9.5 | 9.4 |
| PEM_39[54] | 94.12 | 85.71 | 3 | 62.2 | 10.6 | 7.8 | 11 | 7.9 | 8 | 7.4 |
| PEM_40[55] | 76.92 | 60.00 | 2 | 58.1 | 14.2 | 11.7 | 9 | 9.4 | 13.4 | 15.8 |
| PEM_41[56] | 100.00 | 100.00 | 5 | 65.4 | 11.2 | 8.5 | 10 | 8 | 9.6 | 10.6 |
| PEM_42[57] | 81.82 | 0.00 | 4 | 53.5 | 12.2 | 9.3 | 13 | 8.9 | 10.4 | 8.3 |
| PEM_43[58] | 100.00 | 100.00 | 4 | 64.1 | 11.1 | 8.6 | 10 | 8 | 9.7 | 9.4 |
| PEM_44[12] | 64.71 | 42.86 | 3 | 54.9 | 15.6 | 12.8 | 8 | 9.8 | 14 | 17.4 |
| PEM_45[59] | 91.67 | 60.00 | 3 | 66.3 | 9.1 | 7.7 | 1- | 6.6 | 8.5 | 7.7 |
| PEM_46[60] | 92.31 | 60.00 | 3 | 76.2 | 7.3 | 5.5 | 8 | 5.5 | 5.4 | 5.6 |
| PEM_47[61] | 91.67 | 60.00 | 3 | 55.6 | 12.5 | 11.2 | 10 | 8.8 | 12.3 | 13.7 |
| PEM_48[62] | 93.75 | 100.00 | 4 | 68.5 | 8.7 | 6.9 | 10 | 6.5 | 6.8 | 6.6 |
| PEM_49[63] | 87.50 | 100.00 | 4 | 66.5 | 9.2 | 7.4 | 10 | 6.8 | 7.9 | 7.2 |
| PEM_50[64] | 84.62 | 60.00 | 2 | 64.8 | 9.2 | 7.5 | 11 | 6.8 | 7.8 | 6.9 |
| PEM_51[65] | 84.62 | 66.67 | 4 | 59.7 | 9.4 | 8.1 | 12 | 7.5 | 8.8 | 7 |
| PEM_52[66] | 70.59 | 42.86 | 3 | 50.2 | 13 | 10.8 | 12 | 10.1 | 11.3 | 12.2 |
| PEM_53[67] | 68.75 | 0.00 | 2 | 43.4 | 13.2 | 11.2 | 14 | 9.9 | 12.1 | 11 |
| PEM_54[68] | 68.75 | 60.00 | 2 | 64.7 | 8.9 | 7.5 | 10 | 6.8 | 7.7 | 6.8 |
| PEM_55[69] | 81.82 | 60.00 | 2 | 49.6 | 13.5 | 11 | 12 | 10.2 | 12 | 12.3 |
| PEM_56[70] | 93.33 | 80.00 | 2 | 48.6 | 11.4 | 9.8 | 12 | 8.4 | 9.3 | 7.7 |
| PEM_57[71] | 76.92 | 60.00 | 4 | 48.3 | 14.6 | 12.7 | 10 | 10.3 | 13.6 | 15.7 |
| PEM_58[72] | 100.00 | 100.00 | 5 | 66.5 | 9.7 | 8.2 | 8 | 7 | 7.7 | 8.8 |
| PEM_59[73] | 100.00 | 100.00 | 4 | 80.6 | 6.6 | 4.4 | 8 | 5.1 | 4.1 | 4.5 |
| PEM_60[74] | 92.31 | 80.00 | 3 | 77.2 | 8 | 6.3 | 9 | 6.2 | 8 | 7.7 |
| PEM_61[75] | 93.75 | 80.00 | 3 | 63.9 | 9.5 | 7.2 | 11 | 7.2 | 7.6 | 6.2 |
| PEM_62[76] | 92.86 | 66.67 | 4 | 66.3 | 10.6 | 7.7 | 9 | 7.9 | 7.8 | 8.3 |
| PEM_63[77] | 92.31 | 60.00 | 5 | 66.2 | 10.1 | 7.3 | 11 | 7.6 | 8 | 7.3 |
| PEM_64[78] | 93.33 | 40.00 | 3 | 49.2 | 14.5 | 11.4 | 12 | 10.7 | 12.7 | 13.5 |
| PEM_65[79] | 93.33 | 100.00 | 4 | 65.5 | 11.5 | 8.4 | 9 | 8.4 | 9.1 | 10.7 |
| PEM_66[80] | 100.00 | 60.00 | 3 | 61.7 | 10 | 8.9 | 10 | 7.5 | 9.7 | 9.2 |
| PEM_67[81] | 92.31 | 60.00 | 3 | 55.9 | 13 | 10.4 | 11 | 9.4 | 11.4 | 12.5 |
| PEM_68[82] | 100.00 | 80.00 | 4 | 81.3 | 8.2 | 6 | 7 | 5.4 | 6.2 | 7.9 |
| PEM_69[83] | 92.31 | 60.00 | 4 | 62.4 | 12 | 8.9 | 9 | 8.7 | 9 | 11 |
| PEM_70[84] | 100.00 | 100.00 | 5 | 62.2 | 10.6 | 7.8 | 11 | 7.9 | 8 | 7.4 |
| PEM_71[85] | 100.00 | 100.00 | 3 | 50.3 | 15 | 12.7 | 12 | 10.8 | 15.1 | 16.5 |
| PEM_72[86] | 91.67 | 60.00 | 2 | 69.1 | 9.1 | 7 | 11 | 6.8 | 8.1 | 7.2 |
| PEM_73[87] | 83.33 | 40.00 | 1 | 50.5 | 13.1 | 10.8 | 11 | 9.9 | 11.1 | 12.1 |
| PEM_74[88] | 100.00 | 100.00 | 3 | 76.8 | 8.2 | 6.1 | 8 | 5.8 | 6.4 | 7.1 |
| PEM_75[89] | 90.91 | 42.86 | 3 | 41.8 | 14.4 | 11.9 | 13 | 10.6 | 12.4 | 12.3 |
| PEM_76[90] | 93.75 | 40.00 | 4 | 55.4 | 11 | 9.4 | 12 | 8.2 | 10.1 | 8.7 |
| PEM_77[91] | 86.67 | 57.14 | 4 | 58.2 | 11.1 | 9.3 | 11 | 8 | 9.8 | 9.3 |
| PEM_78[92] | 76.92 | 40.00 | 2 | 54.4 | 10.6 | 11 | 12 | 8.3 | 12.7 | 12.6 |
| PEM_79[93] | 73.33 | 20.00 | 3 | 73.4 | 7.9 | 6.7 | 9 | 5.5 | 7.2 | 7.1 |
| PEM_80[94] | 76.92 | 60.00 | 2 | 46.9 | 12.6 | 10.2 | 13 | 9.1 | 10 | 8.4 |
| PEM_81[95] | 87.50 | 80.00 | 5 | 63.1 | 10.5 | 9.8 | 9 | 7 | 11.1 | 12.3 |
| PEM_82[96] | 64.71 | 42.86 | 3 | 65.4 | 10.3 | 8.1 | 9 | 7.9 | 8.1 | 8.8 |
| PEM_83[97] | 100.00 | 100.00 | 5 | 67.8 | 9.9 | 7.3 | 10 | 7.5 | 7.5 | 7.6 |
| PEM_84[98] | 81.82 | 40.00 | 2 | 53.5 | 14 | 11.7 | 11 | 10.2 | 13.3 | 15 |
| PEM_85[99] | 83.33 | 60.00 | 3 | 65.6 | 11.2 | 7.6 | 10 | 8.4 | 8.3 | 8.2 |
| PEM_86[100] | 75.00 | 60.00 | 2 | 61.9 | 12 | 7.5 | 11 | 8.7 | 7.3 | 7.1 |
| PEM_87[101] | 80.00 | 83.33 | 2 | 59.4 | 12.4 | 8.7 | 13 | 9.1 | 10.9 | 8.9 |
| PEM_88[102] | 94.44 | 100.00 | 5 | 70.6 | 9.5 | 7.5 | 8 | 7 | 7.8 | 8.7 |

**PEMAT-P - Patient Education Material Evaluation Tool for Print Materials ratings, ARI - Automated Readability Index, Lin – Linsear Write Formula, Col - Coleman Liau, Fle - Flesch-Kincaid Grade Level, FRE - Flesch Reading Ease*

*^#^The result is a number that corresponds to the grade level unless for FRE.*

*^&^DISCERN classification: 1 – Low (serious or extensive shortcomings), 2 – Low-Moderate, 3 – Moderate (Potentially important but not serious shortcomings), 4 – Moderate-High, 5 – High (Minimal shortcomings)*

**Table 5.** Summary of Included Patient Decision Aids (n=5)

| ID | International Patient Decision Aids Standards scores | |
| --- | --- | --- |
|  | Certification criteria | Quality criteria |
| PEM_10[26] | 55 | 53.26 |
| PEM_21[37] | 50 | 46.73 |
| PEM_24[40] | 75 | 55.43 |
| PEM_31[47] | 65 | 70.54 |
| PEM_88[102] | 62.5 | 83.04 |

**References**

1. Joseph-Williams N, Newcombe R, Politi M, Durand MA, Sivell S, Stacey D, O'Connor A, Volk RJ, Edwards A, Bennett C *et al*: **Toward Minimum Standards for Certifying Patient Decision Aids: A Modified Delphi Consensus Process**. *Med Decis Making* 2014, **34**(6):699-710.

2. Ouzzani M, Hammady H, Fedorowicz Z, Elmagarmid A: **Rayyan—a web and mobile app for systematic reviews**. *Systematic Reviews* 2016, **5**(1):210.

3. Higgins J, Savović J, Page M, Elbers R, Sterne J: **Chapter 8: Assessing risk of bias in a randomized trial**. In: *Cochrane Handbook of Systematic Review for Interventions.* edn.: Cochrane Collaboration; 2019.

4. **The Newcastle-Ottawa Scale (NOS) for assessing the quality of nonrandomised studies in meta-analyses** [<http://www.ohri.ca/programs/clinical_epidemiology/oxford.asp>]

5. **Cochrane Handbook for Systematic Reviews of Interventions version 6.2** Cochrane; 2021.

6. Bonner C, Patel P, Fajardo MA, Zhuang R, Trevena L: **Online decision aids for primary cardiovascular disease prevention: systematic search, evaluation of quality and suitability for low health literacy patients**. *BMJ Open* 2019, **9**(3):e025173.

7. **Scraper Plugin - Google Chrome** [<https://chrome.google.com/webstore/detail/scraper/mbigbapnjcgaffohmbkdlecaccepngjd?hl=en>]

8. Santos PJF, Daar DA, Badeau A, Leis A: **Readability of online materials for Dupuytren's contracture**. *J Hand Ther* 2018, **31**(4):472-479.

9. Shoemaker SJ, Wolf MS, Brach C: **Development of the Patient Education Materials Assessment Tool (PEMAT): a new measure of understandability and actionability for print and audiovisual patient information**. *Patient Educ Couns* 2014, **96**(3):395-403.

10. Charnock D, Shepperd S: **Learning to DISCERN online: applying an appraisal tool to health websites in a workshop setting**. *Health Educ Res* 2004, **19**(4):440-446.

11. El-Gendy SR: **Controlling dyspnea in chronic obstructive pulmonary disease patients**. *J Egypt Public Health Assoc* 2015, **90**(2):58-63.

12. Qian MYY, Politis J, Thompson M, Wong D, Le B, Irving L, Smallwood N: **Individualized breathlessness interventions may improve outcomes in patients with advanced COPD**. *Respirology* 2018, **23**(12):1146-1151.

13. Apps LD, Mitchell KE, Harrison SL, Sewell L, Williams JE, Young HM, Steiner M, Morgan M, Singh SJ: **The development and pilot testing of the self-management programme of activity, coping and education for chronic obstructive pulmonary disease (SPACE for COPD)**. *Int J Chron Obstruct Pulmon Dis* 2013, **8**:317-327.

14. Wongpiriyayothar A, Piamjariyakul U, Williams PD: **Effects of patient teaching, educational materials, and coaching using telephone on dyspnea and physical functioning among persons with heart failure**. *Appl Nurs Res* 2011, **24**(4):e59-66.

15. Howard C, Dupont S: **'The COPD breathlessness manual': a randomised controlled trial to test a cognitive-behavioural manual versus information booklets on health service use, mood and health status, in patients with chronic obstructive pulmonary disease**. *NPJ Prim Care Respir Med* 2014, **24**:14076.

16. Thomas M, Bruton A, Little P, Holgate S, Lee A, Yardley L, George S, Raftery J, Versnel J, Price D *et al*: **A randomised controlled study of the effectiveness of breathing retraining exercises taught by a physiotherapist either by instructional DVD or in face-to-face sessions in the management of asthma in adults**. *Health Technol Assess* 2017, **21**(53):1-162.

17. **Breathlessness: Fact Sheet 2 - Breathing Methods and Positions to Ease Breathlessness** [<https://www.iow.nhs.uk/Downloads/Patient%20Information%20Leaflets/Breathlessness%20Methods%20and%20positions%20to%20ease%20breathlessness.pdf>]

18. **Breathlessness: Fact Sheet 4 - Breathlessness, Stress and Anxiety** [<https://www.iow.nhs.uk/Downloads/Patient%20Information%20Leaflets/Breathlessness%20stress%20and%20anxiety%20V1.pdf>]

19. **Management of Breathlessness** [<https://edu.cdhb.health.nz/Patients-Visitors/patient-information-pamphlets/Documents/Management-of-Breathlessness-3078.pdf>]

20. **Dyspnea** [<https://www.healthline.com/health/dyspnea>]

21. **Information for Patient and relatives about breathlessness** [<https://www.uhcw.nhs.uk/download/clientfiles/files/Patient%20Information%20Leaflets/Medicine/Palliative%20Care/Understanding%20Breathlessness%20Palliative%20Care%20Team%20(2298).pdf>]

22. **Information for Patients with Breathlessness due to Heart Problems** [<https://www.srft.nhs.uk/EasysiteWeb/getresource.axd?AssetID=86349&type=full&servicetype=Inline>]

23. **Breathlessness Management** [<https://www.nhstaysidecdn.scot.nhs.uk/NHSTaysideWeb/idcplg?IdcService=GET_SECURE_FILE&dDocName=PROD_272580&Rendition=web&RevisionSelectionMethod=LatestReleased&noSaveAs=1>]

24. **Coping with breathlessness** [<https://www.cpft.nhs.uk/Documents/Miscellaneous/Coping%20with%20breathlessness%20March%202018.pdf>]

25. **Managing breathlessness 3: Breathing Techniques** [<https://www.guysandstthomas.nhs.uk/resources/patient-information/acute/breathing-techniques.pdf>]

26. **Living well with COPD: Chronic Bronchitis and Emphysema** [<https://www.lcmmg.com/documents/Living-well-with-COPD.pdf>]

27. **Breathlessness** [<https://www.cancercouncil.com.au/lung-cancer/managing-symptoms/shortness-of-breath/>]

28. **Shortness of Breath** [<https://www.uhn.ca/PatientsFamilies/Health_Information/Health_Topics/Documents/How_to_Manage_Shortness_Breath_cco.pdf>]

29. **Abdominal Breathing** [<https://www.guysandstthomas.nhs.uk/resources/patient-information/therapies/abdominal-breathing.pdf>]

30. **Breathing Exercises and Techniques for COPD** [<https://www.copdfoundation.org/About-Us/Who-We-Are/About-The-COPD-Foundation.aspx>]

31. **Chronic Obstructive Pulmonary Disease (COPD) –Helping You Breathe Easier** [<https://sites.google.com/site/nursingstudentsnote/family-blog/diagnostictestandspecimencollection/1445_COPD_Fact_Sheet.pdf?attredirects=0&d=1>]

32. **How to Cope with Being Short of Breath - Positions** [<https://www.acprc.org.uk/Data/Publication_Downloads/GL-01HowtocopewithbeingSOB-positions(1).pdf>]

33. **Breathlessness** [<https://www.healthnavigator.org.nz/health-a-z/b/breathlessness/>]

34. **Breathing Pattern Disorders** [<https://www.uhs.nhs.uk/Media/UHS-website-2019/Patientinformation/Respiratory/Breathing-pattern-disorders-patient-information.pdf>]

35. **Palliative care - Shortness of Breath** [<https://www.mountsinai.org/health-library/special-topic/palliative-care-shortness-of-breath>]

36. **Breathing problems and exercise** [<https://www.betterhealth.vic.gov.au/health/HealthyLiving/breathing-problems-and-exercise>]

37. **Chronic Obstructive Pulmonary Disease (COPD)** [<https://www.thearchmedicalpractice.co.uk/files/2020/05/COPD-patient-booklet.pdf>]

38. **Shortness of Breath** [<https://www.drugs.com/cg/shortness-of-breath-discharge-care.html>]

39. **Hyperventilation Information** [<https://www.mountsinai.org/health-library/symptoms/hyperventilation>]

40. **Breathing Help Decision Aid** [<http://intermountainhealthcare.org/ext/Dcmnt?ncid=529809939>]

41. **Shortness of Breath** [<https://www.cancer.org/treatment/treatments-and-side-effects/physical-side-effects/shortness-of-breath.html>]

42. **Dyspnea/Shortness of Breath** [<https://www.brighamandwomens.org/lung-center/diseases-and-conditions/dyspnea-shortness-of-breath>]

43. **Breathlessness** [<https://healthengine.com.au/info/breathlessness>]

44. **Shortness of Breath** [<https://www.cancercouncil.com.au/cancer-information/advanced-cancer/living-with-advanced-cancer/managing-symptoms/shortness-of-breath/>]

45. **Lung Cancer: Managing Shortness of Breath** [<https://www.urmc.rochester.edu/encyclopedia/content.aspx?ContentTypeID=34&ContentID=21274-1>]

46. **Managing your breathlessness using a handheld fan** [<https://www.guysandstthomas.nhs.uk/resources/patient-information/therapies/physiotherapy/managing-your-breathlessness-using-a-handheld-fan.pdf>]

47. **Better Living with Chronic Obstructive Pulmonary Disease** [<https://lungfoundation.com.au/wp-content/uploads/2018/09/Book-Better-Living-with-COPD-Dec2016.pdf>]

48. **Living with Dyspnea: How to breathe more easily** [<https://blacklungcoe.org/wp-content/uploads/2018/03/Patient-Education-Breathing.pdf>]

49. **Breathlessness - Shortness of Breath** [<https://www.thoracic.org/patients/patient-resources/resources/breathlessness.pdf>]

50. **What to Know About Hyperventilation: Causes and Treatments** [<https://www.healthline.com/health/hyperventilation>]

51. Schneidman A, Reinke L, Donesky D, Carrieri-Kohlman V: **Patient information series. Sudden breathlessness crisis**. *Am J Respir Crit Care Med* 2014, **189**(5):P9-10.

52. **Shortness of Breath** [<https://foundation.chestnet.org/lung-health-a-z/shortness-of-breath/>]

53. **Pursed Lip Breathing [video]** [<https://www.lung.org/lung-health-diseases/lung-disease-lookup/copd/patient-resources-and-videos/pursed-lip-breathing-video>]

54. **Positions to ease breathlessness** [<https://www.guysandstthomas.nhs.uk/resources/patient-information/acute/positions-for-breathlessness.pdf>]

55. **Patient handout for tips for shortness of breath** [<https://ideasforot.com/?page_id=157>]

56. **Managing breathlessness at home during the COVID-19 pandemic** [<https://www.europeanlung.org/assets/files/factsheets/Breathlessness%20during%20COVID-19/KHP-Factsheet%20-%20D05%20Final.pdf>]

57. **Managing Shortness of Breath Near the End of Life** [<https://www.compassus.com/for-caregivers/is-it-the-right-time-for-hospice/managing-shortness-of-breath>]

58. **Leaflet 3: Breathing techniques to ease breathlessness** [<https://buckup-cuh-production.s3.amazonaws.com/documents/PIN0735_leaflet_3_breathing_techniques_to_ease_breathlessness_v8.pdf>]

59. **Hyperventilation: Symptoms, Causes, Treatment, Emergencies** [<https://www.webmd.com/lung/lung-hyperventilation-what-to-do>]

60. **Hyperventilation: Care Instructions** [<https://myhealth.alberta.ca/Health/aftercareinformation/pages/conditions.aspx?hwid=ut2508>]

61. **Hyperventilation/dysfunctional breathing** [<https://www.gloshospitals.nhs.uk/media/documents/Managing_overbreathing_Hyperventilation_dysfunctional_breathing_GHPI0245_02_17.pdf>]

62. **Hyperventilation syndrome** [<http://publicdocuments.sth.nhs.uk/PIL1019.PDF>]

63. **Hyperventilation syndrome** [<https://www.uhb.nhs.uk/Downloads/pdf/PiHyperventilationSyndrome.pdf>]

64. **Hyperventilation Syndrome - West Suffolk Hospital** [<https://www.wsh.nhs.uk/CMS-Documents/Patient-leaflets/Physiotherapy/5268-1HyperventilationSyndrome.pdf>]

65. **Hyperventilation Syndrome & Breathing Control Advice** [<https://www.nwangliaft.nhs.uk/EasySiteWeb/GatewayLink.aspx?alId=9867>]

66. **Hyperventilation Symptoms & Treatment** [<https://atlantasouth.pediatrix.com/body.cfm?id=7&action=detail&AEArticleID=003071&AEProductID=Adam2004_1&AEProjectTypeIDURL=APT_1>]

67. **Hyperventilation** [<https://www.healthnavigator.org.nz/health-a-z/h/hyperventilation/>]

68. **Hyperventilation** [<http://www.sthk.nhs.uk/patients-visitors/Documents/Hyperventilation%20patient%20information%20leaflet.pdf>]

69. **Hyperventilation** [<https://www.hopkinsmedicine.org/health/conditions-and-diseases/hyperventilation>]

70. **Hyperventilation** [<https://www.mottchildren.org/health-library/hypvn>]

71. **Hyperventilation** [<https://www.yeovilhospital.co.uk/wp-content/uploads/2015/07/Hyperventilation.pdf>]

72. **Hyperventilation** [nhsborders.scot.nhs.uk/media/213548/Hyperventilation.pdf]

73. **How to manage your shortness of breath** [<https://www.uhn.ca/PatientsFamilies/Health_Information/Health_Topics/Documents/How_to_Manage_Shortness_Breath_cco.pdf>]

74. **How to breathe when you are short of breath** [<https://medlineplus.gov/ency/patientinstructions/000053.htm>]

75. **Dyspnea** [<https://rayyan.qcri.org/fulltexts/693308>]

76. **Breathlessness and Anxiety** [<https://my.dchs.nhs.uk/Portals/0/Health%20Psychology%20Breathlessness%20and%20Anxiety_1.pdf>]

77. **Breathlessness IMPRESS Tips (BITs) for Patients** [<https://www.respiratoryfutures.org.uk/resources/impress-documents/breathlessness-impress-tips-for-patients/>]

78. **Breathlessness** [<https://lungfoundation.com.au/patients-carers/after-your-diagnosis-title/breathlessness/>]

79. **Breathlessness** [<https://www.blf.org.uk/support-for-you/breathlessness>]

80. **Breathing pattern disorder** [<https://www.worcsacute.nhs.uk/patient-information-and-leaflets/documents/patient-information-leaflets-a-z/2471-breathing-pattern-disorder>]

81. **Breathing Retraining** [<https://www.cci.health.wa.gov.au/-/media/CCI/Mental-Health-Professionals/Anxiety/Anxiety---Information-Sheets/Anxiety-Information-Sheet---08---Breathing-Retraining.pdf>]

82. **Breathing Exercises - Critical Care Recovery** [<https://www.criticalcarerecovery.com/file/GL-02HowtocopewithbeingSOB-breathingex.pdf>]

83. **Breath training exercises** [<https://services.unimelb.edu.au/counsel/resources/guided-exercises/breath-training>]

84. **Better Living with Chronic Obstructive Pulmonary Disease** [<https://lungfoundation.com.au/health-professionals/conditions/copd/for-your-patients/>]

85. **Best Positions to Reduce Shortness of Breath** [<https://lunginstitute.com/blog/best-positions-to-reduce-shortness-of-breath/>]

86. **Best Breathing Exercises for COPD** [<https://lunginstitute.com/blog/best-breathing-exercises-for-copd/>]

87. **Air Hunger: The Surprising Symptom of Anxiety** [<https://www.mytherapyapp.com/blog/anxiety-and-air-hunger>]

88. **A randomised controlled study of the effectiveness of breathing retraining exercises taught by a physiotherapist either by instructional DVD or in face-to-face sessions in the management of asthma in adults** [Journal]

89. **Breathing and motor neurone disease: what you can do (EB6)** [<https://www.mndnsw.asn.au/about-mnd/information/153-managing-with-mnd/living-better/232-breathing-and-motor-neurone-disease-what-you-can-do-eb6.html>]

90. **Breathing Problems in Adults with Neuromuscular Weakness** [<https://www.thoracic.org/patients/patient-resources/resources/neuromuscular-weakness-adult.pdf>]

91. **Breathing Into a Paper Bag for Anxiety: Does It Work?** [<https://www.healthline.com/health/anxiety/breathing-into-a-paper-bag#when-to-talk-with-a-doctor>]

92. **Understanding Hyperventilation Syndrome** [<https://www.fairview.org/patient-education/90732>]

93. **Hospice Handbook** [<https://www.gundersenhealth.org/app/files/public/9250/Hospice-Patient-Education-handbook.pdf>]

94. **Hyperventilation Syndrome** [<http://www.dchs.nhs.uk/assets/public/dchs/services_we_provide/service-directory/our-services/health-psychology/Information-sheets/stress&anxiety/Hyperventilation%20Syndrome.pdf>]

95. **The Active Cycle of Breathing Techniques** [<https://www.acprc.org.uk/Data/Publication_Downloads/GL-05ACBT.pdf>]

96. **Hyperventilation syndrome**

97. **Help With Breathing Decision Aid** [<https://respectingchoices.org/wp-content/uploads/2020/03/Decision_Aid_Help_with_Breathing.pdf>]

98. **Shortness of Breath of Dyspnea** [<https://www.cancer.net/coping-with-cancer/physical-emotional-and-social-effects-cancer/managing-physical-side-effects/shortness-breath-or-dyspnea>]

99. **Breathlessness Support Service** [<https://www.kcl.ac.uk/cicelysaunders/attachments/breathlessness-final/breathlessness-information.pdf>]

100. **Breathlessness and Difficulty Breathing** [<https://patient.info/signs-symptoms/breathlessness-and-breathing-difficulties-dyspnoea>]

101. **Breathlessness** [<https://s3-ap-southeast-2.amazonaws.com/os-data-2/tgh/listingforms/page314/physiotherapy_-_breathlessness.pdf>]

102. **The Use of Intubation and Mechanical Ventilation for Severe Chronic Obstructive Pulmonary Disease (COPD)** [<https://decisionaid.ohri.ca/docs/das/COPD.pdf>]
